# The Association between Macular Thickness and Axial Length in Myopic Eyes

**DOI:** 10.1155/2019/8913582

**Published:** 2019-07-16

**Authors:** Yeon Woong Chung, Moon Young Choi, Jung-sub Kim, Jin-woo Kwon

**Affiliations:** ^1^Department of Ophthalmology and Visual Science, St. Vincent's Hospital, College of Medicine, Catholic University of Korea, Republic of Korea; ^2^B & VIIT Eye Center, Seoul, Republic of Korea

## Abstract

**Purpose:**

To investigate the relationship between macular thickness and axial length (AL) in myopic eyes.

**Methods:**

We included 441 myopic eyes in this study and measured macular thickness at the fovea and in other macular regions, using optical coherence tomography. We got thickness difference indices (TDIs) which by definition are the values of thickness difference obtained by subtracting the foveal thickness from that of each macula sector to evaluate macular contour. We then analyzed the relationships between AL and foveal thickness and AL and the TDIs of each macular sector.

**Results:**

In polynomial regression analyses, foveal thickness slope was relatively flat up to an AL of 25.5 mm and began to rise from 25.5–26.0 mm. The TDIs were also relatively flat up to AL of 25.5mm and started to show steepened negative slopes from around AL of 25.5 mm. When grouping myopia participants as high myopia or non-high myopia based on AL of 25.5mm, all macular indices of the high myopia group showed significant correlation with AL (all p values <0.01). But all indices of non-high myopia group had no significant correlation with AL.

**Conclusions:**

Average macular thickness profiles showed that appreciable changes started at an AL of 25.5mm.

## 1. Introduction

Many studies have reported that the incidence of myopia in Southeast Asia has been increasing [1, 2]. Myopia is associated with pathological retinal conditions such as macular and retinal degeneration, foveoschisis, macular hole, and rhegmatogenous retinal detachment [3, 4]. The prevalence of these complications has reportedly been increasing among highly myopic patients [4]. However, few studies have explored the degree of myopia required to induce retinal changes [5, 6].

Although retinal changes occur due to mechanical stretching caused by axial elongation [7], many studies have used the spherical equivalent (SE) of the refractive error to define the degree of myopia [8, 9]. Even studies defining myopia based on axial length have used different definitions of high myopia. Some studies defined high myopia as an axial length > 25.0 mm [10, 11], while others defined it by an axial length of > 26.00 mm or 26.5 mm [6, 12, 13].

Therefore, the objective of this study was to define high myopia better by using polynomial regression analyses to characterize associations between macular profiles and axial length.

## 2. Methods

The medical records of all patients with myopia, defined as SE ≤ -0.5 diopters (D), who received a preoperative examination for refractive surgery, were retrospectively reviewed. This study adhered to the tenets of the Declaration of Helsinki, and the study protocol was approved by the institutional review and ethics boards of the Catholic University of Korea.

All patients underwent a full ophthalmic examination that included measurements of visual acuity (VA), refraction, intraocular pressure (IOP), and a fundus examination after achieving maximum pupil dilatation. Macular thickness was measured using optical coherence tomography (OCT) (3D OCT Maestro; Topcon, Tokyo, Japan), and axial length was measured using an IOL Master (Carl Zeiss Meditec, Dublin, CA, USA).

We obtained macular thickness by dividing the macular area into the fovea and inner superior macular, inner nasal macular, inner inferior macular, inner temporal macular, outer superior macular, outer nasal macular, outer inferior macular, and outer temporal sectors by the OCT system of ETDRS map [14].

**Figure 1:**
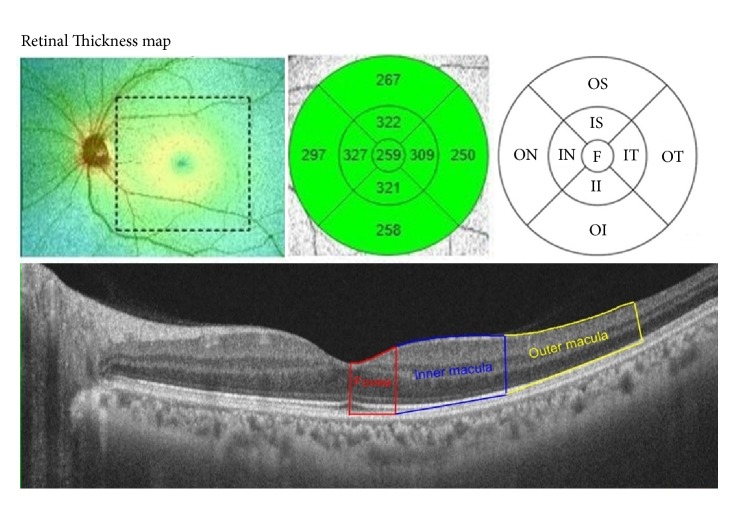
An example and a schematic diagram of a macular optical coherence tomography (OCT) scan. The macular thickness is measured by 256 x 256 (vertical x horizontal) axial scans in the macular region. Macula is divided into 3 concentric rings: the innermost 1.0-mm diameter ring represents the fovea, 3.0mm diameter ring represents the inner macular area, and the outermost 6.0mm diameter ring represents the outer macular area. The value of each sector is reported as averages from each measurement. We additionally obtained thickness difference indices which are the values of thickness differences obtained by subtracting the foveal thickness from that of other macular sectors to identify contour of macula. F, fovea; S, superior; IS, inner superior; OS, outer superior; IN, inner nasal; ON, outer nasal; II, inner inferior; OI, outer inferior; IT, inner temporal; OT, outer temporal.

Protocol of macular thickness measurements consists of 256 x 256 (vertical x horizontal) axial scans in the macular region. Macula is divided into 9 regions with 3 concentric rings: The innermost 1.0mm diameter ring represents the fovea, the 3.0mm diameter ring represents the inner macula, and the outermost 6.0mm ring represents the outer macula (Figure 1). The macular thicknesses of every sector by ETDRS map were averaged for the purpose of analysis.

We additionally obtained thickness difference indices (TDIs) which are the values of thickness differences obtained by subtracting the foveal thickness from that of other macular sectors to determine relative differences in the thickness of the macula with fovea. [e.g., TDI value of inner superior macula (*μ*m) = thickness of inner superior macula (*μ*m) – foveal thickness (*μ*m)].

To reduce any effect of age-related retinal changes, we enrolled patients aged 20–40 years. The inclusion criteria were myopic eyes without glaucomatous disc changes or pathological retinal lesions, such as a lacquer crack, Fuchs' spot, or other markers of retinal degeneration. Patients having eyes with concurrent diseases other than refractive error and those with a best-corrected VA < 20/20, IOP > 21 mmHg, and history of severe ocular trauma, uveitis, intraocular surgery, diabetes, or other vitreoretinal diseases in either eye were excluded.

### 2.1. Statistical Analyses

Statistical analyses were performed using SPSS for Windows (ver. 21.0; SPSS Inc., Chicago, IL, USA) and R software (ver. 3.2.3; R Development Core Team, Vienna, Austria).

We used Pearson's correlation and polynomial regression to determine the association between axial length and macular profile. The statistical significance level was set at p < 0.05.

## 3. Results

We enrolled 441 myopic eyes of 441 patients; one eye per one patient was selected randomly. There were 243 males and 198 females, and their mean age was 26.70 ± 6.19 years. Average of axial length and spherical equivalent was 25.58 ± 1.14mm and -4.73 ± 2.31 diopters, respectively.

**Figure 2:**
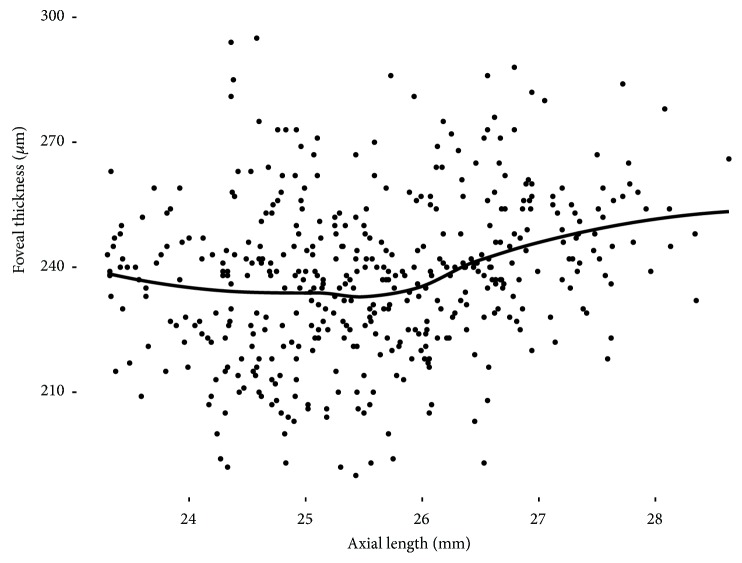
The relationship between foveal thickness and axial length in polynomial regression. Foveal thickness began to increase at an axial length of 25.5–26.0 mm. The grey area denotes the 95% confidence band. F, fovea.

**Figure 3:**
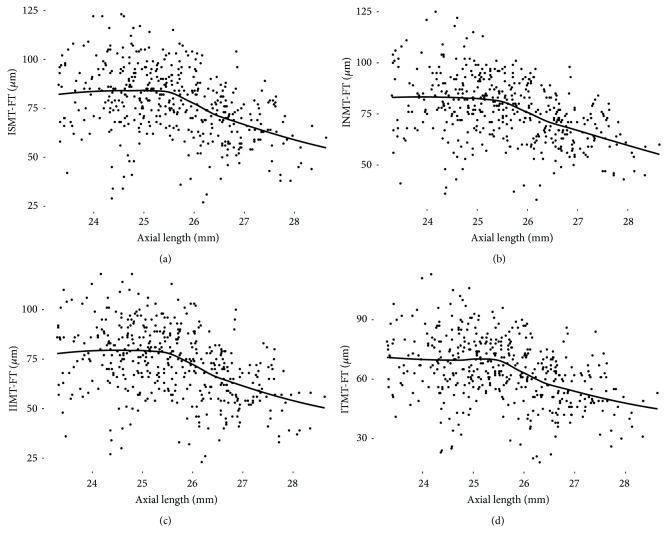
The relationship between axial length and differences in thickness obtained by subtracting the foveal thickness from that of inner macular sectors in polynomial regression analyses. The trend is relatively flat at axial lengths of up to 25.5 mm and started to show steepened negative slopes from around axial length of 25.5 mm. The grey area denotes the 95% confidence band. F, fovea; ISM, inner superior macula; INM, inner nasal macula; IIM, inner inferior macula; ITM, inner temporal macula.

**Figure 4:**
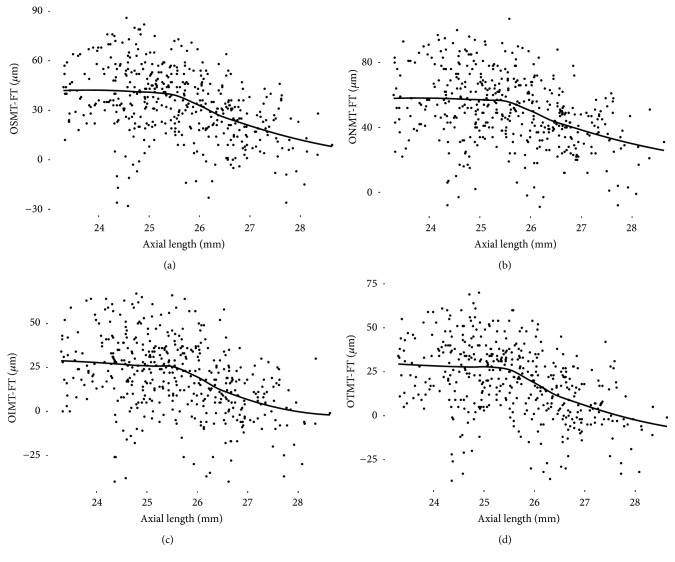
The relationship between axial length and differences in thickness obtained by subtracting the foveal thickness from that of outer macular sectors in polynomial regression analyses. The trend is relatively flat at axial lengths of up to 25.5 mm and started to show steepened negative slopes from around axial length of 25.5 mm. The grey area denotes the 95% confidence band. F, fovea; OSM, outer superior macula; ONM, outer nasal macula; OIM, outer inferior macula; OTM, outer temporal macula.

In polynomial regression analyses, foveal thickness slope was relatively flat at axial lengths of up to 25.5 mm and then began to rise at 25.5–26.0 mm (Figure 2). An observed trend is that the TDIs of inner and outer macular sectors were also relatively flat at axial lengths of up to 25.5 mm and started to show steepened negative slopes from around axial length of 25.5 mm. (Figures 3 and 4).

With these results, we grouped the participants as either high myopia or non-high myopia based on 25.5mm [15] and performed correlation analyses in each group.

In the correlation analyses of non-high myopia group, axial length had no significant correlations with foveal thickness and TDIs of all sectors (Table 1). On the other hand, in the same analysis of the high myopia group, axial length was significantly correlated with foveal thickness and TDIs of all sectors (all p values<0.01). The correlation between axial length and foveal thickness was positive. However, TDIs of all other macular sectors showed negative correlations with axial length (Table 2).

## 4. Discussion

We performed this study to investigate the association between macular thickness and axial length. The results show that there was a range in which macular profiles changed dramatically with axial elongation.

Before the advent of OCT, myopic changes in the macula were thought to be due to atrophy of retinal pigment epithelium at the posterior poles [16]. However, recent OCT studies have reported that the foveal thickness of myopic patients is higher than that of nonmyopic eyes and increases with the progression of myopia [17, 18]. Some studies have suggested that increased axial length causes mechanical stretching of the sclera at the posterior pole, which can induce vitreal traction on the fovea, making it thicker [19]. Another study suggested that foveal reconstruction due to retinal stretching occurred in response to ocular growth in myopic eyes. As a result of foveal reconstruction, the parafovea, which is more elastic, becomes thinner [20]. Our study also found that the fovea was thicker and the parafovea and perifovea were thinner with longer axial length. Although we did not suggest the values of slope in the correlation analysis with high myopic group in the Results Section, the slopes of inner macula ranged from -8.11 to 9.74, and those of outer macula ranged from -10.66 to -11.29. This may mean that the outer macula thickness and contour could be affected more by axial elongation in high myopic eyes with axial length of 25.5mm or more.

Some reports have found that macular thickness differed by sex, race, and age [21, 22]. These studies just compared macular thickness values of each sector. Previous methods could have limitations on identifying changes of macular curvature or contour with axial elongation. In this study, we offer new indices that are values of thickness differences based on the fovea. We suggest that these values can reduce the confounding effect of differences in sex, race, and age. And these relative thickness values based on fovea could be helpful in identifying macular contour.

High myopia has been defined based on the complications arising from axial elongation, or based on optimization of intraocular lens diopter calculations [23–25]. Therefore, the definition of high myopia differs among studies [6, 11–13]. We suggest that the point of onset of anatomical changes in the macula should be determined and also provide new evidence of high myopia. Previous studies investigated differences in macular profiles according to axial length using only linear correlation analyses [17, 18, 20], but no study has been performed to identify the inflection point for changes in macular thickness according to the degree of myopia. In this study, the inflection points of foveal thickness and macular indices occurred at around axial length of 25.5 mm. Because this study to determine changes in macular thickness with axial length was only a pilot investigation, the results should be confirmed by additional studies.

There were some limitations to this study. First, the sample size was too small to obtain significant differences among subgroups of participants classified according to axial length. Second, a more detailed OCT protocol would be helpful for analyzing and identifying changes in the macula. We are planning a follow-up study including a population of myopic patients and additional analyses of OCT findings to address these limitations.

In conclusion, mean foveal thickness did not appreciably increase until an axial length of 25.5 mm, with greater thickness beginning at 25.5–26.0 mm. The parafoveal and perifoveal thicknesses, based on foveal thickness, tended to decrease with axial elongation, and the slope became steeper around axial length of 25.5mm.

## Figures and Tables

**Table 1:** Results of a Pearson correlation of the variables of interest in non-high myopic group (r-values, p-values, and two-sided).

Variable (n=215)	Mean ± SD	AL	F	ISM-F	INM-F	IIM-F	ITM-F	OSM-F	ONM-F	OIM-F
AL (mm)	24.61 ± 0.58									
F (*μ*m)	234.31 ± 19.37	-0.055 (0.418)								
ISM-F (*μ*m)	83.66 ± 16.83	0.028 (0.687)	-0.623*∗*							
INM-F (*μ*m)	82.72 ± 15.38	-0.011 (0.878)	-0.495*∗*	0.941*∗*						
IIM-F (*μ*m)	79.03 ± 16.74	0.013 (0.848)	-0.598*∗*	0.948*∗*	0.934*∗*					
ITM-F (*μ*m)	69.78 ± 15.95	0.009 (0.899)	-0.583*∗*	0.922*∗*	0.890*∗*	0.917*∗*				
OSM-F (*μ*m)	41.34 ± 19.96	-0.013 (0.848)	-0.792*∗*	0.849*∗*	0.725*∗*	0.802*∗*	0.772*∗*			
ONM-F (*μ*m)	57.34 ± 21.07	-0.015 (0.825)	-0.728*∗*	0.865*∗*	0.797*∗*	0.844*∗*	0.777*∗*	0.921*∗*		
OIM-F (*μ*m)	26.60 ± 19.60	-0.044 (0.519)	-0.776*∗*	0.834*∗*	0.742*∗*	0.846*∗*	0.791*∗*	0.919*∗*	0.928*∗*	
OTM-F (*μ*m)	27.75 ± 18.99	-0.007 (0.915)	-0.713*∗*	0.835*∗*	0.724*∗*	0.821*∗*	0.847*∗*	0.873*∗*	0.817*∗*	0.886*∗*

AL, axial length; F, fovea; ISM, inner superior macula; INM, inner nasal macula; IIM, inner inferior macula; ITM, inner temporal macula; OSM, outer superior macula; ONM, outer nasal macula; OIM, outer inferior macula; OTM, outer temporal macula; SD, standard deviation.

*∗*p<0.001.

**Table 2:** Results of a Pearson correlation of the variables of interest in high myopic group (r-values, p-values, and two-sided).

Variable (n=226)	Mean ± SD	AL	F	ISM-F	INM-F	IIM-F	ITM-F	OSM-F	ONM-F	OIM-F
AL (mm)	26.50 ± 0.70									
F (*μ*m)	241.14 ± 18.14	0.330*∗*								
ISM-F (*μ*m)	72.01 ± 15.94	-0.426*∗*	-0.681*∗*							
INM-F (*μ*m)	71.25 ± 13.46	-0.420*∗*	-0.592*∗*	0.924*∗*						
IIM-F (*μ*m)	66.88 ± 16.56	-0.411*∗*	-0.677*∗*	0.948*∗*	0.918*∗*					
ITM-F (*μ*m)	58.67 ± 15.11	-0.385*∗*	-0.675*∗*	0.944*∗*	0.890*∗*	0.947*∗*				
OSM-F (*μ*m)	26.54 ± 18.51	-0.405*∗*	-0.788*∗*	0.902*∗*	0.794*∗*	0.859*∗*	0.841*∗*			
ONM-F (*μ*m)	44.25 ± 19.82	-0.375*∗*	-0.732*∗*	0.895*∗*	0.821*∗*	0.865*∗*	0.821*∗*	0.923*∗*		
OIM-F (*μ*m)	13.07 ± 19.77	-0.388*∗*	-0.806*∗*	0.864*∗*	0.781*∗*	0.864*∗*	0.844*∗*	0.926*∗*	0.921*∗*	
OTM-F (*μ*m)	12.34 ± 18.86	-0.418*∗*	-0.765*∗*	0.899*∗*	0.810*∗*	0.896*∗*	0.900*∗*	0.904*∗*	0.844*∗*	0.902*∗*

AL, axial length; F, fovea; ISM, inner superior macula; INM, inner nasal macula; IIM, inner inferior macula; ITM, inner temporal macula; OSM, outer superior macula; ONM, outer nasal macula; OIM, outer inferior macula; OTM, outer temporal macula; SD, standard deviation.

*∗*p<0.001.

## Data Availability

The data used to support the findings of this study are available from the corresponding author upon request.
